# Molecular pathways involved in the synergistic interaction of the PKC*β* inhibitor enzastaurin with the antifolate pemetrexed in non-small cell lung cancer cells

**DOI:** 10.1038/sj.bjc.6604566

**Published:** 2008-08-19

**Authors:** C Tekle, E Giovannetti, J Sigmond, J R Graff, K Smid, G J Peters

**Affiliations:** 1 Department of Medical Oncology, VU University Medical Center, Amsterdam, The Netherlands; 2 Division of Pharmacology and Chemotherapy, Department of Internal Medicine, University of Pisa, Italy; 3 Eli Lilly & Co, Indianapolis, IN, USA

**Keywords:** enzastaurin, protein kinase C*β*, pemetrexed, cell signalling, non-small cell lung cancer

## Abstract

Conventional regimens have limited impact against non-small cell lung cancer (NSCLC). Current research is focusing on multiple pathways as potential targets, and this study investigated molecular mechanisms underlying the combination of the PKC*β* inhibitor enzastaurin with the multitargeted antifolate pemetrexed in the NSCLC cells SW1573 and A549. Pharmacologic interaction was studied using the combination-index method, while cell cycle, apoptosis induction, VEGF secretion and ERK1/2 and Akt phosphorylation were studied by flow cytometry and ELISAs. Reverse transcription–PCR, western blot and activity assays were performed to assess whether enzastaurin influenced thymidylate synthase (TS) and the expression of multiple targets involved in cancer signaling and cell cycle distribution. Enzastaurin-pemetrexed combination was highly synergistic and significantly increased apoptosis. Enzastaurin reduced both phosphoCdc25C, resulting in G2/M checkpoint abrogation and apoptosis induction in pemetrexed-damaged cells, and GSK3*β* and Akt phosphorylation, which was additionally reduced by drug combination (−58% in A549). Enzastaurin also significantly reduced pemetrexed-induced upregulation of TS expression, possibly through E2F-1 reduction, whereas the combination decreased TS *in situ* activity (>50% in both cell lines) and VEGF secretion. The effects of enzastaurin on signaling pathways involved in cell cycle control, apoptosis and angiogenesis, as well as on the expression of genes involved in pemetrexed activity provide a strong experimental basis to their evaluation as pharmacodynamic markers in clinical trials of enzastaurin-pemetrexed combination in NSCLC patients.

Non-small cell lung cancer (NSCLC) is one of the most commonly occurring malignancies worldwide for which platinum-based regimens are standard first-line treatment (Buter and Giaccone, 2005). However, the dose-limiting toxicity profile of these regimens, as well as response rates not exceeding 40%, warrant novel strategies and new combination regimens against NSCLC.

The multitargeted antifolate pemetrexed is approved as a single agent in second-line treatment of patients with locally advanced or metastatic NSCLC after earlier chemotherapy (Hanna *et al*, 2004). It inhibits three of the enzymes essential for purine and pyrimidine synthesis; dihydrofolate reductase (DHFR), glycinamide ribonucleotide formyl transferase, and thymidylate synthase (TS), the latter being the most important target (Hanauske *et al*, 2001). However, resistance to pemetrexed may develop (Peters *et al*, 2005), and hence combinations with other anticancer agents are important to investigate.

Enzastaurin, a novel targeted agent, selectively inhibits PKC*β* by interacting competitively at its ATP-binding site (Faul *et al*, 2003). Because of its pivotal role in the regulation of tumour-induced angiogenesis, cell cycle progression, tumour cell proliferation, survival, and tumour invasiveness, PKC*β* is recognised as an important target for cancer treatment (Goekjian and Jirousek, 2001; Liu *et al*, 2004). Enzastaurin was originally evaluated in human tumour xenograft-bearing mice for its antiangiogenic activity upon PKC*β* inhibition, as it showed reduction of plasma VEGF levels together with a significant decrease in intratumoural vessel density (Keyes *et al*, 2004). However, several studies have shown that enzastaurin exhibits direct growth inhibiting effects on a wide array of cultured human tumour cells (Graff *et al*, 2005; Oberschmidt *et al*, 2005; Querfeld *et al*, 2006; Rizvi *et al*, 2006; Podar *et al*, 2007; Spalding *et al*, 2007; Lee *et al*, 2008). Recent studies suggest that the antitumour effects of enzastaurin are mediated through interference with the phosphatidylinositol 3-kinase (PI3K)/Akt pathway (Graff *et al*, 2005; Querfeld *et al*, 2006; Rizvi *et al*, 2006; Lee *et al*, 2008), an important pathway regulating the apoptotic response.

The advantage of enzastaurin and other targeted agents is that they can act selectively on inappropriately expressed or overexpressed molecules in cancer signaling pathways. Overexpression of different PKC isoforms has been detected in NSCLC cells and tumour tissues (Clark *et al*, 2003; Lahn *et al*, 2006), whereas activation of Akt was associated with significantly worse 5-year survival rate in NSCLC patients (Tang *et al*, 2006).

Currently enzastaurin is being evaluated in several clinical trials and tolerability and survival data obtained in a recent phase II trial as second- or third-line therapy in NSCLC suggest further evaluation in combination with cytotoxic drugs (Oh *et al*, 2008). Indeed other studies have demonstrated the safety of enzastaurin combination with conventional chemotherapy (Hanauske *et al*, 2006; Rademaker-Lakhai *et al*, 2007), and the inhibition of selected targets, including PKC*β*, can enhance the effect of cytotoxic drugs, such as pemetrexed. Previous studies showed positive synergism for this combination in thyroid and lung cancer cell lines (Oberschmidt *et al*, 2005; Nakajima *et al*, 2006), but data on possible molecular mechanisms or biomarkers of that combination are still lacking.

The aim of this study was to investigate the main pharmacological aspects of the enzastaurin-pemetrexed combination in NSCLC cells. For this purpose the potential synergistic interaction, as well as the responsible mechanisms were studied.

## Materials and methods

### Drugs and chemicals

Both pemetrexed and enzastaurin were provided by Eli Lilly Corporation (Indianapolis, IN, USA). The drugs were dissolved in Hanks' balanced salt solution and Dimethyl Sulphoxide (DMSO) respectively, stored at −20°C, and diluted in culture medium immediately before use. (5-^3^H)-Deoxycytidine and (5-^3^H)-DeoxyUMP were from Moravek Biochemicals (Brea, CA, USA). All other chemicals were of analytical grade.

### Cells and culture conditions

The NSCLC cell lines A549 (adenocarcinoma) and SW1573 (alveolar carcinoma) were from American Type Culture Collection (ATCC) (Manassas, VA, USA), and were cultured in Dulbecco's modified Eagle's medium (Flow Laboratories Irvine, Scotland), supplemented with 10% heat-inactivated Fetal calf serum, 20 mM 2-(4-(2-Hydroxyethyl)-1-piperazinyl)-ethanesulphonic acid, and 1% penicillin–streptomycin (Gibco Paisley, UK), at 37°C under an atmosphere of 5% CO_2_. The cells were maintained in 75 cm^2^ tissue culture flasks (Greiner Bio-One GmbH, Frickenhausen, Germany) and were harvested with trypsin-EDTA (Invitrogen, Paisley, UK) in their exponentially growing phase.

### Growth inhibition and drug combination studies

Growth inhibitory effects of pemetrexed and enzastaurin were evaluated with the MTT (3-(4,5-dimethylthiazol-2-yl)-2,5-diphenyltetrazolium bromide) assay, which measures mitochondrial activity of viable cells. both cell lines (3000 cells per well) were plated into flat bottom 96-well plates (Greiner Bio-One GmbH, Frickenhausen, Germany) and allowed to attach for 24 h. The cells were treated for 72 h as follows: (1) a concentration range (0.01–50 *μ*M) of the indicated drugs to determine the IC_50_ values; (2) simultaneous combination of the two drugs using a fixed IC_50_-based molar ratio; (3) simultaneous combination using a variable ratio (with constant IC_25_ concentration of enzastaurin); (4) sequential combination using a variable ratio. After treatment, the medium was removed and cells were incubated for 3 h at 37°C in 50 *μ*l per well MTT solution (final concentration 0.42 mg ml^−1^). Formazan crystals formed were dissolved in 150 *μ*l per well DMSO and the absorbance was measured at 540 nm using a spectrophotometric microplate reader (Tecan Spectrafluor, Salzburg, Austria). Absorbance values were corrected for the absorbance at the day of drug administration, which was taken as 0%, the difference with untreated controls was set at 100%, and the effect of drugs as a percentage thereof. The 50% inhibitory concentration of cell growth was estimated from the logarithmic growth inhibition curves.

The pharmacological interaction of enzastaurin and pemetrexed was assessed using the multiple drug effect analysis based on the methods described by Chou and Talalay (1984) in which a Combination Index (CI)<0.9, 0.9–1.1 and >1.1 indicate synergism, additivity and antagonism, respectively. The mean CI was calculated from all data points with fraction affected (FA)>0.5, as values lower were not considered relevant for growth inhibition (Peters *et al*, 2000). The data were processed by the Calcusyn Software (Biosoft, Cambridge, UK), which calculates the CI of the combination based on the effect of the growth inhibition caused by the drugs alone relative to the effect produced by the combination.

### Cell cycle and apoptosis analysis

Flow cytometry was used to determine cell cycle distribution as well as the amount of apoptotic cells within the cell populations exposed to the drugs alone or in combination as described previously (Temmink *et al*, 2007). Cells at a density of 50 × 10^3^ cells per well were plated into flat bottom 6-well plates (Greiner Bio-One GmbH, Frickenhausen, Germany) and allowed to attach for 24 h prior to drug treatments at IC_50_ concentrations. The exposure time ranged from 24–72 h involving both single drug treatments as well as simultaneous combination drug treatments. Subsequently, adherent and floating cells were harvested and counted, and at a concentration of 1 × 10^5^ cells ml^−1^ transferred to round-bottom FALCON tubes (BD, Franklin Lakes, NJ, USA). After centrifugation, the cell pellets formed were gently resuspended in 1.0 ml hypotonic propidium iodide (PI)-solution (50 *μ*g ml^−1^ PI, 0.1% sodium citrate, 0.1% Triton X-100, 0.1 mg ml^−1^ ribonuclease A) and the samples were stored on ice for 30 min. Cell cycle analyses were performed using a FACScan (BD Biosciences, Mount View, CA, USA) and the data analysis was carried out with CELLQuest™ software, using gates on DNA histograms to estimate the amount of cells in G1, S, and G2/M phases, as well as the apoptotic cells in the sub-G1 region.

### Real-time PCR

To evaluate the effect of 24-h treatment with IC_50_ levels of enzastaurin, pemetrexed and their combination on the expression levels of molecules involved in drug activity, cell cycle control and angiogenesis we studied the mRNA expression of TS, E2F-1 and VEGF. RNA was extracted by the QiaAmp RNA mini-Kit (Qiagen, San Diego, CA, USA), and reverse transcribed. Forward and reverse primers and probes were designed with Primer Express (Applied Biosystems, Foster City, CA, USA) on the basis of *TS* gene sequence (Giovannetti *et al*, 2005), whereas primers and probes for *E2F-1*, and *VEGF* were obtained from Applied Biosystems Assay-on-Demand Gene expression products (Hs001572991_m1, and Hs00173626_m1). Amplification data were normalised to *β*-actin, and quantification of gene expression was performed using standard curves obtained with dilutions of cDNA from Quantitative-PCR Human-Reference Total-RNA (Stratagene, La Jolla, CA, USA).

### Western blot analysis

Enzastaurin and pemetrexed and their combination were also studied for their ability to modulate protein expression of different possible targets or surrogate markers of drug activity by western blot analyses. Frozen pellets of A549 and SW1573 cells previously treated with IC_50_ concentrations of pemetrexed and enzastaurin (single and in the simultaneous combination) for 24 h were resuspended in a lysis buffer (0.1% (v/v) Triton X-100, 10% glycerol, 150 mM NaCl, 10 mM Tris-HCL pH 7.6, 50 mM *β*-glycerophosphate, and 5 mM EDTA). Protein content was assessed using the BioRad assay, whereas SDS–PAGE was performed as described earlier (Sigmond *et al*, 2003). Briefly, whole cell lysates were denatured in sample buffer containing SDS and *β*-mercaptoethanol, and equal amounts of total protein were separated on 10–15% SDS–poly-acrylamide gels and transferred to polyvinylidene fluoride membranes. After blocking with 5% nonfat dry milk/TBST, the membranes were incubated overnight at 4°C with the primary antibodies diluted in 5% BSA/TBST. The following antibodies were used: anti-phospho-Akt (Ser473) 1 : 1000, anti-Akt 1 : 1000, anti-MAPK 1 : 1000, anti-phospho-GSK3*β* (Ser9) 1 : 1000, anti-GSK3*β* 1 : 1000, anti-phospho-Cdc25C (Ser216) 1 : 1000, anti-Cdc25C 1 : 1000, anti-phospho-CDK2 (Thr 160) 1 : 1000, anti-CDK2 1 : 1000, anti-CDK4 1 : 2000 (all from Cell Signaling Technology Inc., Danvers, MA, USA), anti-E2F-1 1 : 100, anti-PKCβ 1 : 500, anti-COX-2 1 : 2000 (all from Santa Cruz Biotechnology Inc., Santa Cruz, CA, USA), anti-TS 1 : 1000 (Provided by Dr GW Aherne, Institute for Cancer Research, Sutton, UK) (van Triest *et al*, 2000), and as a loading control anti-*β*-actin 1 : 10 000 (Sigma-Aldrich, St Louis, MO, USA). The subsequent day, the membranes were incubated with the appropriate horseradish peroxidase-conjugated secondary antibodies, and detection was performed using chemiluminescence reagent (GE Healthcare Bio-Sciences Corp, Piscataway, NJ, USA) according to the manufacturer's protocol.

### ERK1/2 and Akt phosphorylation assays

To study the effect of drug treatments on the activation of ERK1/2 and Akt, cells were exposed to IC_50_ s of enzastaurin, pemetrexed and enzastaurin-pemetrexed combination for 24 h. After protein extraction from cell pellets, the dual-phosphorylation of ERK2 at threonine 185 and tyrosine 187 (ERK2 (pTpY185/187)) and ERK1 at threonine 202 and tyrosine 204 (ERK1 (pTpY202/204)) and Akt phosphorylation at serine residue 473 (Akt (pS473)), were evaluated with specific ELISA assays (BioSource International, Camarillo, CA, USA), and normalised respectively to the total EGFR, ERK1/2 and Akt and protein content (Giovannetti *et al*, 2008).

### VEGF levels

Measurement of VEGF levels in medium was performed after exposing 1 × 10^5^ cells to IC_50_ concentrations of the drugs alone and in combination. Samples of the medium (200 *μ*l) were taken after 24 h, centrifuged for 20 min at 1000 **g** and immediately frozen until analysis. Vascular endothelial growth factor levels were measured using a specific ELISA kit (R&D diagnostics, Minneapolis, USA) according to the instructions of the manufacturer. A calibration line was included in each plate.

### Evaluation of TS activity

To evaluate the possible modulation of TS *in situ* activity, we determined its potential inhibition in intact cells, after 24 h drug exposure at IC_50_ s. For this purpose cells were plated at 0.25 × 10^6^ cells in 6-well plates. After 22 h of drug treatment (5-^3^H)-deoxycytidine (0.3 *μ*M, final specific activity 1.6 Ci mmol^−1^) was added. After uptake, (5-^3^H)-deoxycytidine was phosphorylated to (5-^3^H)-dCMP, which was deaminated to (5-^3^H)-dUMP, which was in turn methylated to dTMP releasing ^3^H_2_O. Production of ^3^H_2_O was measured by collecting medium samples after 2 h and counting of the radioactivity as described (Rots *et al*, 1999). Furthermore, to study the direct effect of enzastaurin and pemetrexed on TS we also measured TS catalytic activity in extracts of SW1573 and A549 cells at 1 or 10 *μ*M (5-^3^H)dUMP, as described earlier (Sigmond *et al*, 2003).

## Results

### Growth inhibition studies of pemetrexed and enzastaurin

**Figure 1:**
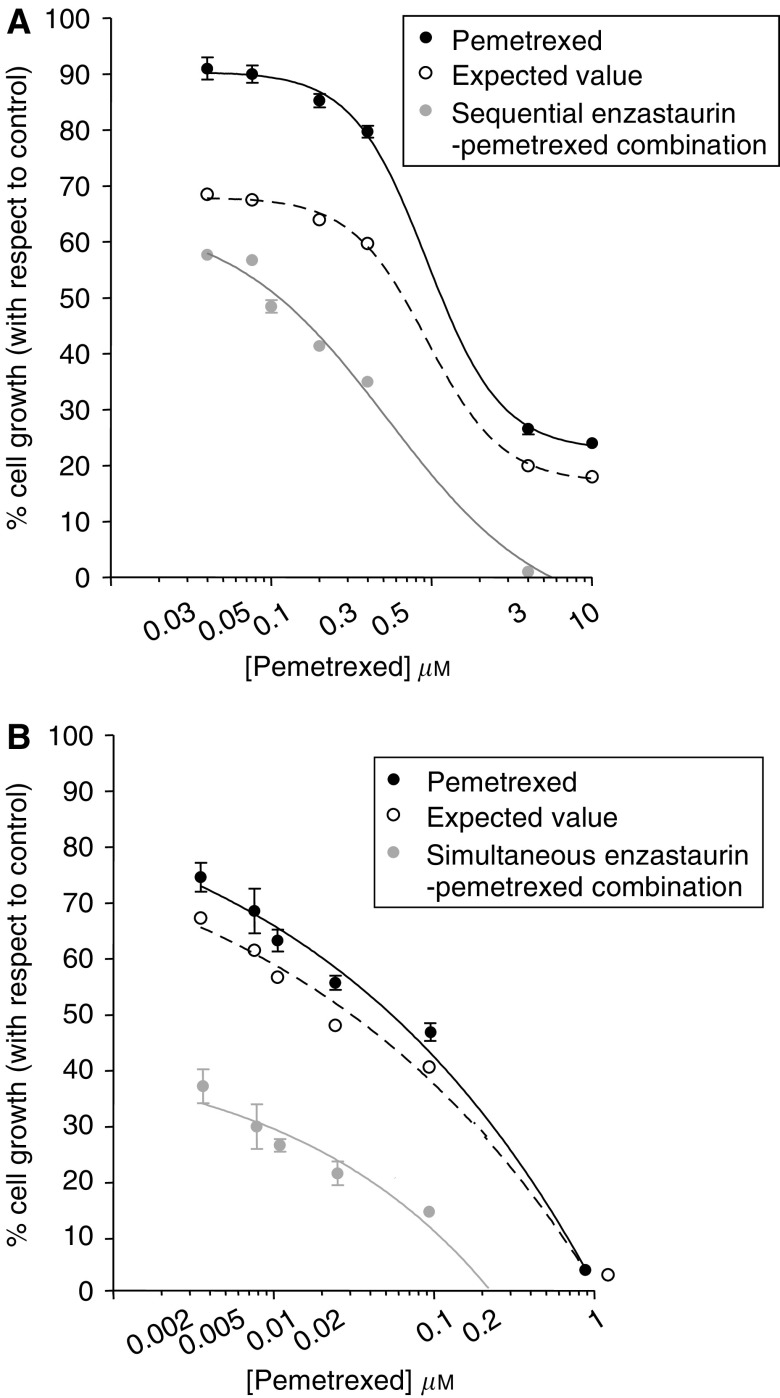
Cytotoxicity and pharmacological interaction of enzastaurin and pemetrexed. Representative curves of growth inhibitory effects of pemetrexed and sequential (24-h enzastaurin followed by 48-h pemetrexed+enzastaurin exposure) drug combination (**A**) and simultaneous enzastaurin-pemetrexed combination (**B**) in SW1573 cells, using a variable ratio (with constant IC_25_ concentration of enzastaurin). Points and columns, mean values obtained from three independent experiments; bars, s.e.

A dose-dependent inhibition of cell growth was observed with pemetrexed and enzastaurin (Figure 1), with IC_50_ values of 0.05 and 21.75 *μ*M (SW1573) and 0.19 and 8.83 *μ*M (A549), respectively (Table 1). As the CI method recommends a ratio of concentrations at which drugs are equipotent, the following combination studies were performed using fixed ratios with IC_50_ values calculated from the previous cytotoxicity analysis for the different drug treatments in each cell line (i.e., 1 : 50 and 1 : 450 in A549 and SW1573 cells, respectively). However, as enzastaurin is administered orally (Carducci *et al*, 2006) and reaches a relatively stable plasma concentration for a prolonged time, we also used enzastaurin at a fixed IC_25_ concentration in simultaneous combination in both cell lines and in the sequential combination in the less sensitive SW1573 cells.

Both the simultaneous and the sequential combination reduced the IC_50_ values of pemetrexed in the studied cell lines. Representative growth inhibition curves for SW1573 cells are shown in Figure 1A and B. The multiple drug effect analysis revealed strong synergistic effects in the simultaneous treatment as well as in the sequential schedule. In particular, the CI plots of simultaneous combination in both cells showed a clear synergistic interaction at the more relevant FA values (⩾50%). The average CI values for enzastaurin-pemetrexed combinations in the two NSCLC cell lines are summarised in Table 1.

To evaluate the mechanisms underlying the synergistic interaction in both cell lines, several biochemical analyses were performed with the simultaneous combination, as detailed below.

### Cell cycle distribution

**Figure 2:**
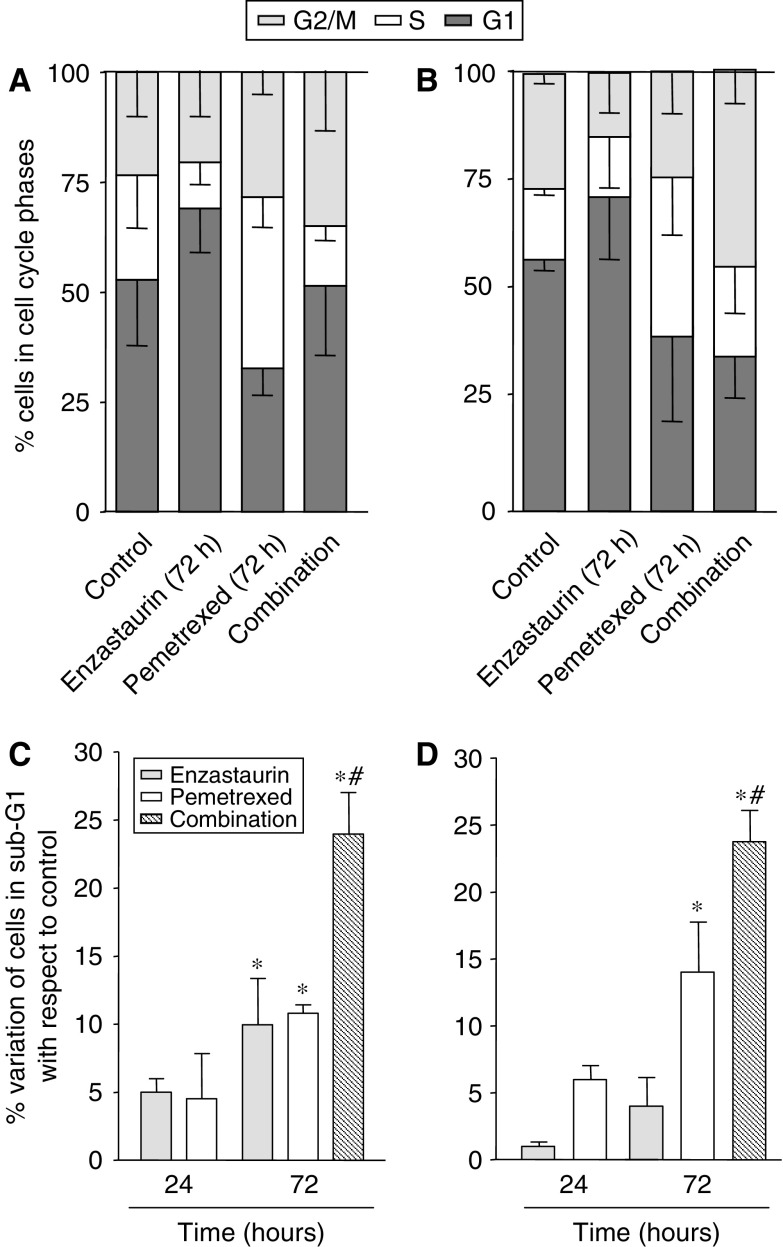
Cell cycle modulation and apoptosis induction. Effects of enzastaurin, pemetrexed and their simultaneous combination on the cell cycle distribution in SW1573 (**A**) and A549 cells (**B**) after 72 h exposure to their IC_50_ values. Apoptosis (%) induced by enzastaurin, pemetrexed and the simultaneous combination in SW1573 (**C**) and A549 (**D**) cells after 24 and 72 h time points. Values (%) are means from three independent experiments±s.e.m. Columns (%), mean values obtained from three independent experiments; bars, s.e.; ^*^P<0.05 with respect to control cells, ^#^*P*<0.05 with respect to pemetrexed-treated cells.

DNA flow cytometry studies were performed to evaluate the effect of enzastaurin, pemetrexed and their combinations on the cell cycle distribution and to determine whether their cell cycle modulating activity might provide clues to optimise drug scheduling. Both agents were able to affect the cell cycle of the studied NSCLC cells (Figure 2A and B).

Pemetrexed treatment resulted in a 1.3 to 1.9-fold increase in the percentage of cells in the S-phase after 24 h in SW1573 and A549 cells, respectively. The increment in S-phase was most pronounced after 72 h, with 37 and 39% of the SW1573 and A549 cells arrested in the S-phase. In contrast, the 24 h enzastaurin treatment caused minimal perturbations in the A549 cells, and a 1.5-fold increase in the percentage of SW1573 cells in the G2/M phase. In both cell lines enzastaurin led to 1.3-fold increase in the G1 phase at 72 h. However, the simultaneous combination at 72 h resulted in a G2/M phase increase in both cell lines. In particular, the increase in the G2/M phase cell population was most pronounced in A549 cells (from 25 to 43% in control and treated cells respectively), whereas SW1573 cells showed a 1.5-fold enhancement.

### Apoptosis

The extent of apoptosis induction was investigated as the accumulation of cells with sub- G1 DNA content in FACS analysis, and was time-dependent in both cell lines, with minimal changes at 24 h. After 72 h enzastaurin slightly increased the percentage of apoptotic cells in SW1573 cells (Figure 2C), whereas a significant increase with respect to control was observed in A549 cells (Figure 2D). Pemetrexed induced more apoptosis than enzastaurin in both cell lines, ranging from 10.8 to 14.0% in SW1573 and A549 cells, respectively (*P*<0.01 *vs* control). The combination showed a more than additive cell kill with respect to the single drugs and a significant induction in apoptosis compared with both controls and pemetrexed-treated cells (*P*<0.001 *vs* control and *P*<0.05 *vs* pemetrexed).

### Modulation of signal transduction

**Figure 3:**
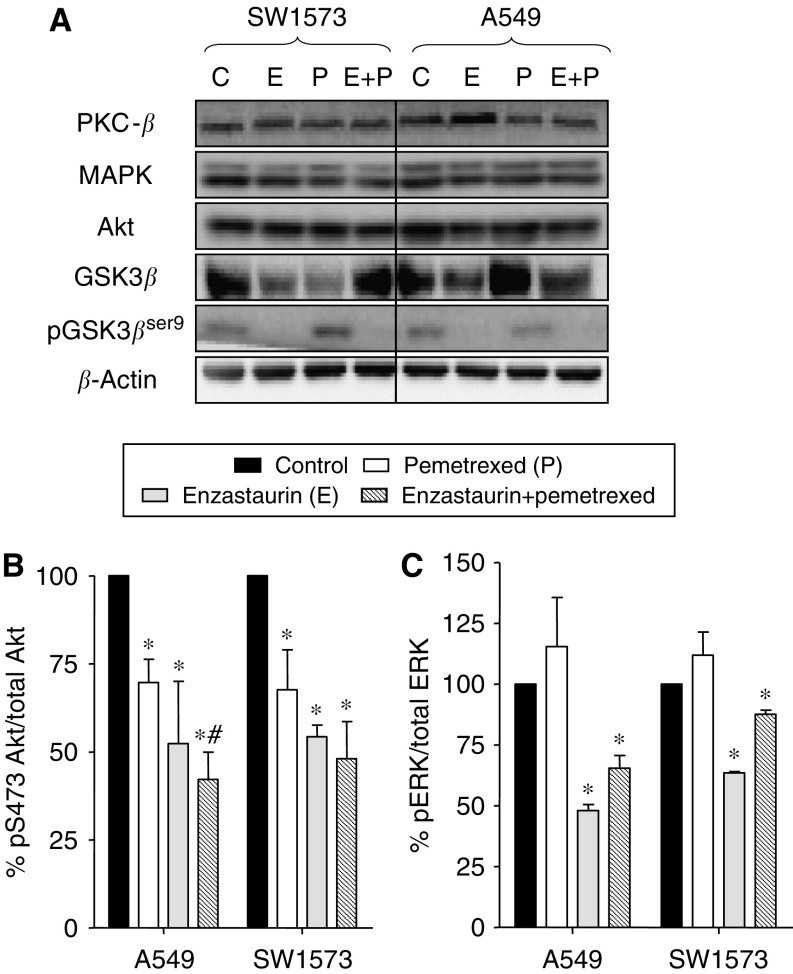
Modulation of cell signaling pathways. (**A**) Modulation of protein expression of different targets involved in cell signaling pathways by enzastaurin, pemetrexed and their simultaneous combination (24 h to IC_50_ values). The western blots shown are representative of 2–3 separate experiments, loading 20 *μ*g protein. Modulation of Akt (**B**), and ERK1/2 (C) phosphorylation by enzastaurin, pemetrexed and their simultaneous combination, for 24 h, at IC_50_ values, in NSCLC cells, as determined with ELISA assays. Columns, mean values obtained from three independent experiments; bars, s.e. ^*^Significantly different from controls (*P*<0.05).

Since enzastaurin affects several intracellular signaling cascades, we initially focused on expression of different proteins downstream of PKC*β* (Figure 3A). Western blot analyses did not show significant modulation in the expression of PKC*β* in both A549 and SW1573 cells treated with enzastaurin, pemetrexed and their simultaneous combination. Similarly total MAPK and total Akt were not affected by drug treatments. However, the expression of the target downstream of Akt, GSK3*β* was reduced by enzastaurin in both cell lines. In contrast, pemetrexed reduced GSK3*β* expression in the SW1573 cells whereas an unexpected relevant increase with respect to control was observed in A549 cells. However, the enzastaurin-pemetrexed combination resulted in a slight reduction of GSK3*β* expression in A549 cells. Furthermore, enzastaurin completely suppressed the phosphorylation of GSK3*β* in both cell lines. Likewise, GSK3*β*^ser9^ was inhibited by the combination even to undetectable levels (Figure 3A).

Phosphorylation of GSK3*β*^ser9^ has been repeatedly linked to Akt activity. Indeed the suppression of GSK3*β*^ser9^ may reflect the inhibition of PKCs as well as of the Akt pathway. Consistent with the latter possibility, the ELISA assays showed that enzastaurin significantly reduced the Akt phosphorylation at the serine residue pS473, and therefore the phospho-Akt/total Akt ratio, in both cell lines (Figure 3B). A lower, but still significant decrease in phospho-Akt was also induced by pemetrexed. Finally, Akt phosphorylation status was additionally reduced by the simultaneous combination of enzastaurin and pemetrexed, with a degree of inhibition of 68 and 62% in A549 and SW1573 cells, respectively. These data are in agreement with our previous results, showing the highest apoptosis induction in cells treated with drug combination.

PKC activation can also trigger signaling through the ERK pathway, which may also be involved in the control of cellular proliferation and death. Enzastaurin resulted in a significant inhibition of pERK1/2 in both cell lines, whereas pemetrexed slightly increased ERK1/2 phosphorylation (Figure 3B). A reduction of phospho-ERK1/2, less pronounced than the one observed with enzastaurin alone, but still significant with respect to controls, was also detected after the drug combination.

### Modulation of cell cycle proteins and TS

**Figure 4:**
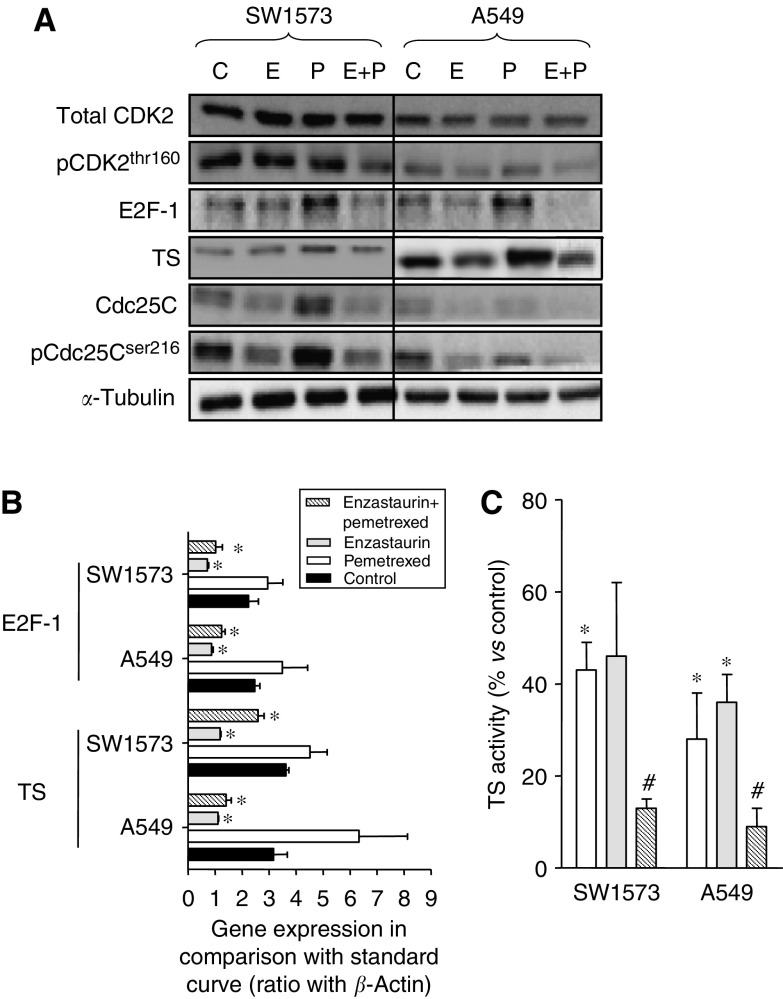
Effects of enzastaurin and pemetrexed on the expression of different targets involved in cell cycle regulation. (**A**) Modulation of protein expression of different targets involved in cell cycle by enzastaurin, pemetrexed and their simultaneous combination (24 h to IC_50_ values); (**B**) Modulation of E2F-1 and TS mRNA expression as determined by real-time PCR; and (**C**) modulation of TS *in situ* activity. The TS *in situ* activity values were calculated as percentages of values obtained in control cells (i.e., 215 and 321 pmol/h/10^6^ cells in A549 and SW1573 cells, respectively). The blots shown are representative of 2–3 separate experiments, loading 20 *μ*g protein. ^*^Significantly different from controls (*P*<0.05). ^#^Significantly different from cells treated with pemetrexed (*P*<0.05).

As PKCs regulate cell cycle progression by phosphorylation (directly or indirectly) of cell cycle-dependent kinases and transcription factors, we evaluated the expression of several of these proteins by western blot analysis (Figure 4A). Our findings suggest that enzastaurin might affect the cell cycle at both the G2/M and G1/S checkpoints. In particular, enzastaurin resulted in a marked decrease of both total and phosphorylated Cdc25C in both cell lines, as shown in a representative example in Figure 4A. The dephosphorylation of Cdc25C induces G2-M transition. Hence, enzastaurin treatment might abrogate the G2/M checkpoint. Consequently, cells with DNA damage (due to pemetrexed treatment) can progress in the cell cycle and eventually undergo apoptosis.

Enzastaurin did not affect CDK2 and CDK4 (data not shown), but was able to downregulate the S-phase regulator E2F-1 and possibly influenced the G1/S checkpoint. Indeed, our results suggest that enzastaurin has a direct effect on E2F-1, which regulate in turn the transcription of several genes, including TS. E2F-1 mRNA expression was significantly reduced by enzastaurin treatment, whereas pemetrexed increased E2F-1 mRNA levels (Figure 4B). However, a significant reduction of E2F-1 mRNA expression was also detected after the drug combination in both cell lines. These results were consistent with those of Figure 4A, showing that pemetrexed increased E2F-1 protein expression, whereas E2F-1 protein levels were significantly reduced by enzastaurin and the enzastaurin-pemetrexed combination.

As TS expression has been correlated with pemetrexed activity both in the preclinical and in the clinical settings, the present study also investigated the changes in TS mRNA and protein expression as well as in TS activity in treated cells. In comparison with the respective controls, both the A549 and the SW1573 cells treated with enzastaurin were characterised by a significant reduction in *TS* mRNA, whereas pemetrexed markedly increased *TS* mRNA (Figure 4B). However, a significant reduction in TS mRNA expression was also detected after enzastaurin-pemetrexed simultaneous combination in A549 cells, whereas a lower degree of inhibition (−28%) was detected in SW1573 cells. Thymidylate synthase expression was also studied at the protein level, by western blotting analysis, which revealed that enzastaurin and pemetrexed affected TS protein expression in both NSCLC cells (Figure 4A). In particular, a strong induction was detected in A549 and SW1573 pemetrexed-treated cells, whereas the faintest bands were observed in the extracts of enzastaurin-treated cells. Furthermore, enzastaurin was able to reduce the upregulation of TS caused by pemetrexed, as detected in the cells treated with the enzastaurin-pemetrexed combination.

As protein expression of TS is not always predictive for the real enzymatic activity in the cells, we then evaluated TS activity by the TS *in situ* assay, in which intact cells are used, and the drugs are still present in the cells, resulting in an actual measurement of real intracellular TS inhibition (Figure 4C). This assay showed a clear inhibition of TS by pemetrexed and enzastaurin in both cell lines. Most interestingly, the combination almost completely inhibited the TS activity *in situ* (i.e., 13±2 and 9±4% in SW1573 and A549 cells, respectively) and statistical analysis revealed significant reductions with respect to those observed after pemetrexed exposure.

Direct inhibition of TS activity by nzastaurin was excluded as addition of enzastaurin up to 50 *μ*M did not inhibit TS activity in extracts of both cell lines, whereas FdUMP and pemetrexed, used as a control, showed similar inhibition as reported earlier (Van Triest *et al*, 1999).

### Effects on COX-2 and VEGF

As enzastaurin has been reported to have an anti-VEGF effect we evaluated the expression of possible markers of antiangiogenic activity both in cells and in cell culture medium.

**Figure 5:**
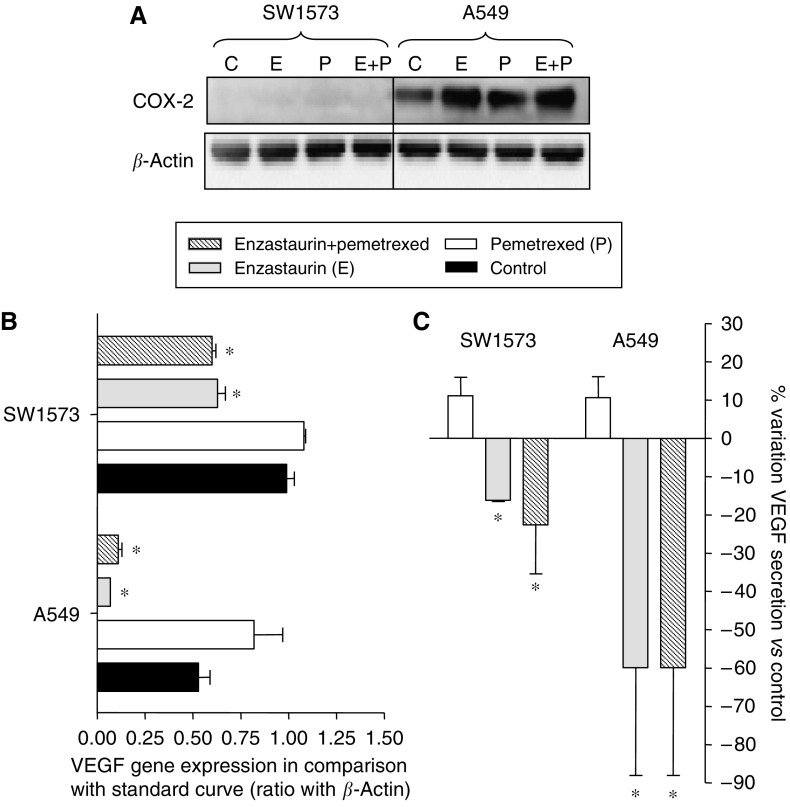
Antiangiogenic effects. Effects of pemetrexed, enzastaurin and their simultaneous combination on COX-2 expression (**A**), as detected by western blot analysis, and on VEGF mRNA expression (**B**) and secretion in cell culture medium (**C**) of SW1573 and A549 cells, as detected by PCR and ELISA assay, respectively. Columns (%), mean values obtained from three independent experiments; bars, s.e.; ^*^P<0.05 with respect to control cells.

In particular, we investigated the expression level of the pro-angiogenic factor COX-2 to determine whether it could serve as a reliable marker of the antiangiogenic effect of enzastaurin in NSCLC cells. However, no expression of COX-2 was found in the SW1573 cell line. Furthermore in the A549 cells the expression levels were still elevated after drug exposure, indicating that the antiangiogenic effects of enzastaurin might not be because of modulation of the COX-2 enzyme in the studied cell lines (Figure 5A).

On the other hand, enzastaurin significantly decreased both VEGF mRNA expression and VEGF secretion into the medium. Pemetrexed induced a slight increase of VEGF expression (Figure 5B and C). However, the combination induced a significant inhibition of VEGF expression. In line with this, VEGF secretion was significantly reduced after enzastaurin-pemetrexed combination in both cell lines (Figure 5C).

## Discussion

In this study we found that the interaction between enzastaurin and pemetrexed was highly synergistic in NSCLC cells. These results are in agreement with previous data obtained with pemetrexed-enzastaurin combination both in lung and in thyroid cancer cells (Oberschmidt *et al*, 2005).

The synergistic activity of enzastaurin was detected in all the studied combinations, at concentrations similar to those achieved in clinical trials (Rademaker-Lakhai *et al*, 2007).

These findings are of importance as pemetrexed is already registered for treatment of NSCLC, and enzastaurin might be a promising agent to improve the effect of pemetrexed in NSCLC patients, possibly as an alternative to cisplatin regimens. Unlike EGFR-tyrosine kinase inhibitors, antiangiogenic agents combined with conventional chemotherapy have demonstrated clinical benefit in NSCLC (Herbst *et al*, 2007), and recent studies have demonstrated the safety of enzastaurin combination with cytotoxic drugs (Rademaker-Lakhai *et al*, 2007), including pemetrexed (Hanauske *et al*, 2006).

**Figure 6:**
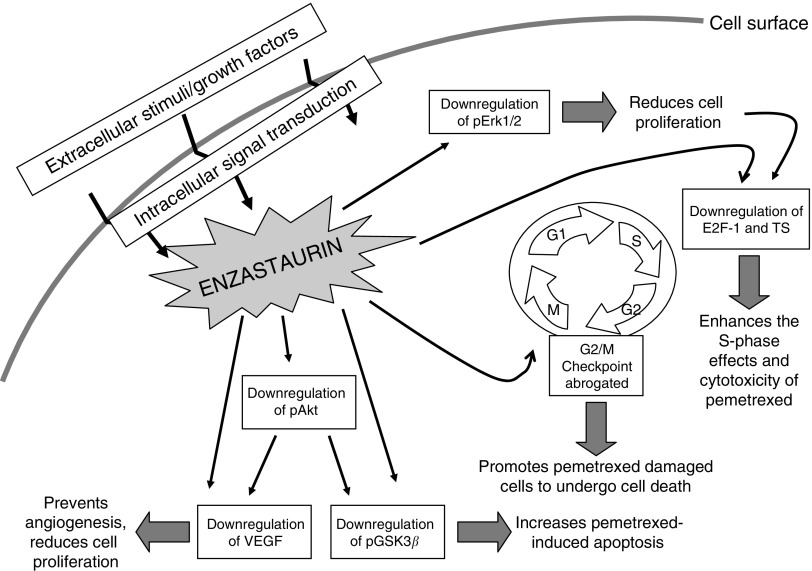
Molecular signaling pathways involved in the synergistic interaction of the PKC*β* inhibitor enzastaurin with pemetrexed. Enzastaurin enhanced the growth inhibitory effects of pemetrexed through its pronounced anti-signaling effects downstream of PKCβ. Moreover, the modulation of cell cycle regulating proteins enhanced both apoptosis induction and pemetrexed-mediated TS enzyme inhibition.

Furthermore, our findings are novel because they show that the synergistic interaction seems to be mediated by several mechanisms, summarised in Figure 6, which enhanced the sensitivity to pemetrexed and should be used as predictive biomarkers for the future clinical development of enzastaurin-pemetrexed combination.

Several studies have shown the importance of modulating the cell cycle to exploit the effect of drug combinations (Schwartz and Shah, 2005). Drugs interacting at different sites in the cell cycle might potentiate the therapeutic response. In this study, FACS analysis demonstrated that pemetrexed caused an S-phase arrest, as previously detected in different tumour cell lines (Tonkinson *et al*, 1997, 1999; Giovannetti *et al*, 2005). In contrast, enzastaurin alone increased the percentage of cells in the G1 phase, whereas in the combination the cells accumulated in G2/M phase. These results are in agreement with previous studies showing that PKCs mediate the regulation of the cell cycle (Fishman *et al*, 1998; Black, 2000). In particular, the phosphorylation mediated by PKC might activate Cdc25C, which is an important control component of the G2/M checkpoint (Yu *et al*, 2004). This inhibits transition into mitosis, as phosphorylated Cdc25C is not able to activate its downstream targets and the cells become arrested at this checkpoint. This arrest prevents DNA damaged cells to cycle further and represents a survival mechanism that provides the tumour cells the opportunity to repair their damaged DNA (Houtgraaf *et al*, 2006). Therefore the reduction of phosphoCdc25C by enzastaurin, as detected by western blot analysis, might explain the abrogation of the G2/M checkpoint and the synergistic induction of apoptosis in the enzastaurin-pemetrexed combination. Indeed, pemetrexed is able to damage the DNA because of incomplete replication (Backus *et al*, 2000), which can lead to arrest of the cells at the checkpoints controlling the cell cycle. Thus, when combining these two agents, enzastaurin can facilitate pemetrexed-damaged cells to undergo apoptosis. In line with our observations, previous studies showed little effects of enzastaurin alone on cell cycle progression (Lee *et al*, 2008), but others reported that the non-selective PKC inhibitor UCN-01 was able to inhibit the phosphorylation of Cdc25C and abrogate the G2/M checkpoint, potentiating the cytotoxicity of a variety of anticancer agents (Yu *et al*, 1998; Graves *et al*, 2000).

However, enzastaurin was also able to influence other proteins involved in cell cycle regulation. In particular, the G1/S checkpoint is, among other proteins, governed by the S-phase regulator E2F-1, whose mRNA and protein expression was significantly reduced by both enzastaurin and enzastaurin-pemetrexed treatment. These results may be related to the inhibition of the Ras/MAPK/ERK-dependent pathway, which is implicated in the modulation of the expression of the cyclin D1 gene. Recent studies showed controversial results on the effects of enzastaurin on ERK pathway in different tumour cell lines (Guo *et al*, 2008; Lee *et al*, 2008). However, cyclin D1 protein expression was reduced after 24 h exposure at IC_50_ values of UCN-01 (Akiyama *et al*, 1997) and cyclin D1 downregulation results in E2F-1 inhibition (Kobayashi *et al*, 2006). Furthermore, UCN-01 induced the accumulation of underphosphorylated pRb (the dephosphorylated retinoblastoma protein form), which prevented the release of free E2F-1 (Akiyama *et al*, 1997).

E2F-1 is a critical upstream transcriptional regulator of several genes, including *TS* and *DHFR* (Li *et al*, 2006). Expression levels of TS were associated with E2F-1 expression in NSCLC cells and samples (Huang *et al*, 2007; Giovannetti *et al*, 2008).

Several studies showed that TS expression is significantly correlated with pemetrexed sensitivity both in the preclinical and in the clinical setting (Giovannetti *et al*, 2005; Gomez *et al*, 2006). Therefore, the reduction of E2F-1 expression, leading to a decreased amount of TS, can potentiate pemetrexed activity in the enzastaurin-treated cells. TS, as an RNA-binding protein, also regulates its own synthesis by impairing the translation of its mRNA, whereas the binding to a specific inhibitor leads to upregulation of TS protein (Chu *et al*, 1991). In agreement with this hypothesis, as well as with the observed increase in *TS* mRNA expression, as previously detected with pemetrexed and 5-fluorouracil (5-FU) (Peters *et al*, 2002; Mauritz *et al*, 2007), TS protein expression in cell extracts was enhanced after pemetrexed exposure. However, this study also shows that enzastaurin-pemetrexed combination significantly inhibited the activity of TS, potentially leading to synergistic drug interaction. These results are in agreement with previous data demonstrating that UCN-01 was able to enhance the activity of 5-FU through downregulation of TS (Hsueh *et al*, 1998; Abe *et al*, 2000).

Enzastaurin also reduced the phosphorylation of the downstream PKC*β*-signaling pathway effectors Akt and GSK3*β*; these findings are consistent with several previous reports in different cancer cell lines, xenografts and peripheral blood mononuclear cells (PBMCs) (Graff *et al*, 2005; Querfeld *et al*, 2006; Rizvi *et al*, 2006), suggesting the use of GSK3*β* phosphorylation in PBMCs as a pharmacodynamic marker for enzastaurin (Graff *et al*, 2005). Our experiments showed that the Akt pathway can also be affected by pemetrexed, as reported previously (Giovannetti *et al*, 2005, 2008). In addition, the enzastaurin-pemetrexed combination significantly reduced Akt phosphorylation, which may explain the increased apoptosis found in the pemetrexed-erlotinib combination in the studied NSCLC cell lines.

Although recent preclinical studies have shown that enzastaurin has a direct effect on several human cancer cells, tumour xenograft models (Graff *et al*, 2005; Lee *et al*, 2008), and also patient-derived tumour explants (Hanauske *et al*, 2007), enzastaurin was initially developed for its striking antiangiogenic activity. In particular, enzastaurin significantly decreased intratumoural vessels density and VEGF expression in different human tumour xenografts (Keyes *et al*, 2004). Similarly, a recent study showed a significant reduction of VEGF protein levels, as well as a reduction of VEGF induction by radiotherapy, in the supernatants of glioma cells exposed for 24 h to enzastaurin (Tabatabai *et al*, 2007). Likewise in our study enzastaurin significantly decreased both VEGF mRNA expression and VEGF secretion into the medium. Furthermore, despite the slight increase of VEGF expression induced by pemetrexed, the enzastaurin-pemetrexed combination resulted in a significant inhibition of VEGF expression, possibly leading to reduced secretion of VEGF. The mechanism of this decrease was postulated to be related to COX-2, as a strong correlation between COX-2 and VEGF mRNA was reported in NSCLC patients (Yuan *et al*, 2005). However, in SW1573 no COX-2 expression was detected, whereas in A549 cells COX-2 levels increased. Hence, VEGF decrease might be related to other factors, as reported earlier in A549 xenograft tumour tissues (Shaik *et al*, 2006). In a clinical study performed in our institution enzastaurin reduced VEGF plasma levels (Peters *et al*, 2007). However, a recent clinical trial in advanced NSCLC patients treated with enzastaurin did not show a consistent change in plasma VEGF levels, but low baseline VEGF levels were associated with longer progression-free survival (Oh *et al*, 2008). Therefore, future studies are warranted to determine whether VEGF levels can be used as a predictive marker of the activity of enzastaurin alone or in combination with other anticancer agents.

Finally, other inhibitors of PKCs, such as staurosporine and its analogue UCN-01, have the same properties as enzastaurin when it comes to inhibition of the cell cycle, but are less selective than enzastaurin. This non-selective inhibition of PKCs makes them too toxic and hence their use in the clinical setting seems limited, whereas enzastaurin which is a selective PKC*β* inhibitor, has shown to be well-tolerated by patients in clinical trials (Carducci *et al*, 2006; Oh *et al*, 2008).

In conclusion, enzastaurin is a very promising anticancer agent, attacking several cellular signaling pathways that are involved in the proliferation, cell cycle control, inhibition of apoptosis and of pro-angiogenic properties of tumour cells. This makes it a good candidate for different combination regimens, including combinations with other novel targeted agents and cytotoxic drugs commonly used in the clinical setting.

## Figures and Tables

**Table 1:** Cytotoxic effect and pharmacological interaction of enzastaurin and pemetrexed in NSCLC cell lines

**IC_50_ (*μ*M)^a^**			**CI^b^**		
**Cell line**	**Enzastaurin**	**Pemetrexed**	**Simultaneous fixed ratio**	**Simultaneous variable ratio**	**Sequential variable ratio**
SW1573	21.75±1.97	0.05±0.1	0.56±0.07	0.09±0.02	0.25±0.035
A549	8.83±1.59	0.19±0.2	0.3±0.06	0.03±0.01	c

a IC_50_ concentrations of the two drugs were calculated as mean values±s.e.m. of at least three independent experiments.

b CI values of simultaneous and sequential enzastaurin-pemetrexed combination at FA of 0.5, 0.75 and 0.9 were averaged for each experiment, and this value was used to calculate the mean between experiments, as described in the Materials and methods section.

c No CI value for this cell line, as A549 cells were less sensitive to pemetrexed compared with the SW1573 cells, and the 48-h exposure to pemetrexed, in the concentration range used in our study, was not enough cytotoxic to allow the correct calculation of the CI values in the enzastaurin (24 h) → pemetrexed+enzastaurin (48 h) sequential schedule.

## References

[bib1] Abe S, Kubota T, Otani Y, Furukawa T, Watanabe M, Kumai K, Kitajima M (2000) UCN-01 (7-hydroxystaurosporine) enhances 5-fluorouracil cytotoxicity through down-regulation of thymidylate synthetase messenger RNA. Jpn J Cancer Res 91: 1192–1198 11092986 10.1111/j.1349-7006.2000.tb00904.x PMC5926291

[bib2] Akiyama T, Yoshida T, Tsujita T, Shimizu M, Mizukami T, Okabe M, Akinaga S (1997) G1 phase accumulation induced by UCN-01 is associated with dephosphorylation of Rb and CDK2 proteins as well as induction of CDK inhibitor p21/Cip1/WAF1/Sdi1 in p53-mutated human epidermoid carcinoma A431 cells. Cancer Res 57: 1495–1501 9108451

[bib3] Backus HH, Pinedo HM, Wouters D, Kuiper CM, Jansen G, Van Groeningen CJ, Peters GJ (2000) Differences in the induction of DNA damage, cell cycle arrest, and cell death by 5-fluorouracil and antifolates. Oncol Res 12: 231–239 11417748 10.3727/096504001108747729

[bib4] Black JD (2000) Protein kinase C-mediated regulation of the cell cycle. Front Biosci 5: D406–D423 10762593 10.2741/black

[bib5] Buter J, Giaccone G (2005) Medical treatment of non-small-cell lung cancer. Ann Oncol 16(Suppl 2): ii229–ii232 15958463 10.1093/annonc/mdi721

[bib6] Carducci MA, Musib L, Kies MS, Pili R, Truong M, Brahmer JR, Cole P, Sullivan R, Riddle J, Schmidt J, Enas N, Sinha V, Thornton DE, Herbst RS (2006) Phase I dose escalation and pharmacokinetic study of enzastaurin, an oral protein kinase C beta inhibitor, in patients with advanced cancer. J Clin Oncol 24: 4092–4099 16943527 10.1200/JCO.2005.05.3447

[bib7] Chou TC, Talalay P (1984) Quantitative analysis of dose-effect relationships: the combined effects of multiple drugs or enzyme inhibitors. Adv Enzyme Regul 22: 27–55 6382953 10.1016/0065-2571(84)90007-4

[bib8] Chu E, Koeller DM, Casey JL, Drake JC, Chabner BA, Elwood PC, Zinn S, Allegra CJ (1991) Autoregulation of human thymidylate synthase messenger RNA translation by thymidylate synthase. Proc Natl Acad Sci USA 88: 8977–8981 1924359 10.1073/pnas.88.20.8977 PMC52634

[bib9] Clark AS, West KA, Blumberg PM, Dennis PA (2003) Altered protein kinase C (PKC) isoforms in non-small cell lung cancer cells: PKCdelta promotes cellular survival and chemotherapeutic resistance. Cancer Res 63: 780–786 12591726

[bib10] Faul MM, Gillig JR, Jirousek MR, Ballas LM, Schotten T, Kahl A, Mohr M (2003) Acyclic N-(azacycloalkyl)bisindolylmaleimides: isozyme selective inhibitors of PKCbeta. Bioorg Med Chem Lett 13: 1857–1859 12749884 10.1016/s0960-894x(03)00286-5

[bib11] Fishman DD, Segal S, Livneh E (1998) The role of protein kinase C in G1 and G2/M phases of the cell cycle (review). Int J Oncol 12: 181–186 9454903 10.3892/ijo.12.1.181

[bib12] Giovannetti E, Lemos C, Tekle C, Smid K, Nannizzi S, Rodriguez JA, Ricciardi S, Danesi R, Giaccone G, Peters GJ (2008) Molecular mechanisms underlying the synergistic interaction of erlotinib, an epidermal growth factor receptor tyrosine kinase inhibitor, with the multitargeted antifolate pemetrexed in non-small-cell lung cancer cells. Mol Pharmacol 73: 1290–1300 18187583 10.1124/mol.107.042382

[bib13] Giovannetti E, Mey V, Nannizzi S, Pasqualetti G, Marini L, Del Tacca M, Danesi R (2005) Cellular and pharmacogenetics foundation of synergistic interaction of pemetrexed and gemcitabine in human non-small-cell lung cancer cells. Mol Pharmacol 68: 110–118 15795320 10.1124/mol.104.009373

[bib14] Goekjian PG, Jirousek MR (2001) Protein kinase C inhibitors as novel anticancer drugs. Expert Opin Investig Drugs 10: 2117–2140 10.1517/13543784.10.12.2117 11772309

[bib15] Gomez HL, Santillana SL, Vallejos CS, Velarde R, Sanchez J, Wang X, Bauer NL, Hockett RD, Chen VJ, Niyikiza C, Hanauske AR (2006) A phase II trial of pemetrexed in advanced breast cancer: clinical response and association with molecular target expression. Clin Cancer Res 12: 832–838 16467096 10.1158/1078-0432.CCR-05-0295

[bib16] Graff JR, McNulty AM, Hanna KR, Konicek BW, Lynch RL, Bailey SN, Banks C, Capen A, Goode R, Lewis JE, Sams L, Huss KL, Campbell RM, Iversen PW, Neubauer BL, Brown TJ, Musib L, Geeganage S, Thornton D (2005) The protein kinase Cbeta-selective inhibitor, Enzastaurin (LY317615.HCl), suppresses signaling through the AKT pathway, induces apoptosis, and suppresses growth of human colon cancer and glioblastoma xenografts. Cancer Res 65: 7462–7469 16103100 10.1158/0008-5472.CAN-05-0071

[bib17] Graves PR, Yu L, Schwarz JK, Gales J, Sausville EA, O'Connor PM, Piwnica-Worms H (2000) The Chk1 protein kinase and the Cdc25C regulatory pathways are targets of the anticancer agent UCN-01. J Biol Chem 275: 5600–5605 10681541 10.1074/jbc.275.8.5600

[bib18] Guo K, Liu Y, Zhou H, Dai Z, Zhang J, Sun R, Chen J, Sun Q, Lu W, Kang X, Chen P (2008) Involvement of protein kinase C beta-extracellular signal-regulating kinase 1/2/p38 mitogen-activated protein kinase-heat shock protein 27 activation in hepatocellular carcinoma cell motility and invasion. Cancer Sci 99: 486–496 18167130 10.1111/j.1349-7006.2007.00702.x PMC11158944

[bib19] Hanauske A, Weigang Koehler K, Yilmaz E, Graefe T, Kuenen B, Thornton D, Lahn M, Darstein C, Musib L, Giaccone G (2006) Comparison of enzastaurin pharmacokinetics and safety in the once daily (QD) and twice daily (BID) dose regimens: A phase I study. *ASCO Annual Meeting Proceedings 2006*. J Clin Oncol 18S: 2047

[bib20] Hanauske AR, Chen V, Paoletti P, Niyikiza C (2001) Pemetrexed disodium: a novel antifolate clinically active against multiple solid tumors. Oncologist 6: 363–373 11524555 10.1634/theoncologist.6-4-363

[bib21] Hanauske AR, Oberschmidt O, Hanauske-Abel H, Lahn MM, Eismann U (2007) Antitumor activity of enzastaurin (LY317615.HCl) against human cancer cell lines and freshly explanted tumors investigated in in-vitro (corrected) soft-agar cloning experiments. Invest New Drugs 25: 205–210 17347872 10.1007/s10637-007-9038-7

[bib22] Hanna N, Shepherd FA, Fossella FV, Pereira JR, De Marinis F, von Pawel J, Gatzemeier U, Tsao TC, Pless M, Muller T, Lim HL, Desch C, Szondy K, Gervais R, Shaharyar, Manegold C, Paul S, Paoletti P, Einhorn L, Bunn Jr PA (2004) Randomized phase III trial of pemetrexed *vs* docetaxel in patients with non-small-cell lung cancer previously treated with chemotherapy. J Clin Oncol 22: 1589–1597 15117980 10.1200/JCO.2004.08.163

[bib23] Herbst RS, O'Neill VJ, Fehrenbacher L, Belani CP, Bonomi PD, Hart L, Melnyk O, Ramies D, Lin M, Sandler A (2007) Phase II study of efficacy and safety of bevacizumab in combination with chemotherapy or erlotinib compared with chemotherapy alone for treatment of recurrent or refractory non small-cell lung cancer. J Clin Oncol 25: 4743–4750 17909199 10.1200/JCO.2007.12.3026

[bib24] Houtgraaf JH, Versmissen J, van der Giessen WJ (2006) A concise review of DNA damage checkpoints and repair in mammalian cells. Cardiovasc Revasc Med 7: 165–172 16945824 10.1016/j.carrev.2006.02.002

[bib25] Hsueh CT, Kelsen D, Schwartz GK (1998) UCN-01 suppresses thymidylate synthase gene expression and enhances 5-fluorouracil-induced apoptosis in a sequence-dependent manner. Clin Cancer Res 4: 2201–2206 9748140

[bib26] Huang CL, Liu D, Nakano J, Yokomise H, Ueno M, Kadota K, Wada H (2007) E2F1 overexpression correlates with thymidylate synthase and survivin gene expressions and tumor proliferation in non small-cell lung cancer. Clin Cancer Res 13: 6938–6946 18056168 10.1158/1078-0432.CCR-07-1539

[bib27] Keyes KA, Mann L, Sherman M, Galbreath E, Schirtzinger L, Ballard D, Chen YF, Iversen P, Teicher BA (2004) LY317615 decreases plasma VEGF levels in human tumor xenograft-bearing mice. Cancer Chemother Pharmacol 53: 133–140 14593497 10.1007/s00280-003-0713-x

[bib28] Kobayashi S, Shimamura T, Monti S, Steidl U, Hetherington CJ, Lowell AM, Golub T, Meyerson M, Tenen DG, Shapiro GI, Halmos B (2006) Transcriptional profiling identifies cyclin D1 as a critical downstream effector of mutant epidermal growth factor receptor signaling. Cancer Res 66: 11389–11398 17145885 10.1158/0008-5472.CAN-06-2318

[bib29] Lahn M, McClelland P, Ballard D, Mintze K, Thornton D, Sandusky G (2006) Immunohistochemical detection of protein kinase C-beta (PKC-beta) in tumour specimens of patients with non-small cell lung cancer. Histopathology 49: 429–431 16978209 10.1111/j.1365-2559.2006.02461.x

[bib30] Lee KW, Kim SG, Kim HP, Kwon E, You J, Choi HJ, Park JH, Kang BC, Im SA, Kim TY, Kim WH, Bang YJ (2008) Enzastaurin, a protein kinase C beta inhibitor, suppresses signaling through the ribosomal S6 kinase and bad pathways and induces apoptosis in human gastric cancer cells. Cancer Res 68: 1916–1926 18339873 10.1158/0008-5472.CAN-07-3195

[bib31] Li W, Xu RJ, Zhang HH, Jiang LH (2006) Overexpression of cyclooxygenase-2 correlates with tumor angiogenesis in endometrial carcinoma. Int J Gynecol Cancer 16: 1673–1678 16884383 10.1111/j.1525-1438.2006.00408.x

[bib32] Liu Y, Su W, Thompson EA, Leitges M, Murray NR, Fields AP (2004) Protein kinase CbetaII regulates its own expression in rat intestinal epithelial cells and the colonic epithelium *in vivo*. J Biol Chem 279: 45556–45563 15322124 10.1074/jbc.M407701200

[bib33] Mauritz R, van Groeningen CJ, Smid K, Jansen G, Pinedo HM, Peters GJ (2007) Thymidylate synthase and dihydropyrimidine dehydrogenase mRNA expression after administration of 5-fluorouracil to patients with colorectal cancer. Int J Cancer 120: 2609–2612 17330233 10.1002/ijc.22626

[bib34] Nakajima E, Helfrich B, Chan D, Zhang Z, Hirsch FR, Chen V, Ma D, Bunn PA (2006) Enzastaurin a protein kinase Cbeta-selective inhibitor, inhibits the growth of SCLC and NSCLC cell lines. *ASCO Annual Meeting Proceedings 2006*. J Clin Oncol 18S: 13138

[bib35] Oberschmidt O, Eismann U, Schulz L, Struck S, Blatter J, Lahn MM, Ma D, Hanauske AR (2005) Enzastaurin and pemetrexed exert synergistic antitumor activity in thyroid cancer cell lines *in vitro*. Int J Clin Pharmacol Ther 43: 603–604 16372535

[bib36] Oh Y, Herbst RS, Burris H, Cleverly A, Musib L, Lahn M, Bepler G (2008) Enzastaurin, an oral serine/threonine kinase inhibitor, as second- or third-line therapy of non-small-cell lung cancer. J Clin Oncol 26: 1135–1141 18309949 10.1200/JCO.2007.14.3685

[bib37] Peters GJ, Backus HH, Freemantle S, van TB, Codacci-Pisanelli G, van der Wilt CL, Smid K, Lunec J, Calvert AH, Marsh S, McLeod HL, Bloemena E, Meijer S, Jansen G, van Groeningen CJ, Pinedo HM (2002) Induction of thymidylate synthase as a 5-fluorouracil resistance mechanism. Biochim Biophys Acta 1587: 194–205 12084461 10.1016/s0925-4439(02)00082-0

[bib38] Peters GJ, Hooiberg JH, Kaspers GJL, Jansen G (2005) Folates and antifolates in the treatment of cancer; role of folic acid supplementation on efficacy of folate and non-folate drugs. Trends in food science and technology 16: 289–297

[bib39] Peters GJ, Tekle C, Kuenen B, Honeywell RJ, Sigmond J, Giovannetti E, Hanauske AR, Lahn M, Giaccone G (2007) A translational study on the protein kinase C-β inhibitor enzastaurin and the thymidylate synthase inhibitor pemetrexed. *AACR Annual Meeting Proceedings 2007*, abstract **1820**

[bib40] Peters GJ, Van der Wilt CL, van Moorsel CJ, Kroep JR, Bergman AM, Ackland SP (2000) Basis for effective combination cancer chemotherapy with antimetabolites. Pharmacol Ther 87: 227–253 11008002 10.1016/s0163-7258(00)00086-3

[bib41] Podar K, Raab MS, Zhang J, McMillin D, Breitkreutz I, Tai YT, Lin BK, Munshi N, Hideshima T, Chauhan D, Anderson KC (2007) Targeting PKC in multiple myeloma: *in vitro* and *in vivo* effects of the novel, orally available small-molecule inhibitor enzastaurin (LY317615. HCl). Blood 109: 1669–1677 17023575 10.1182/blood-2006-08-042747 PMC1794057

[bib42] Querfeld C, Rizvi MA, Kuzel TM, Guitart J, Rademaker A, Sabharwal SS, Krett NL, Rosen ST (2006) The selective protein kinase C beta inhibitor enzastaurin induces apoptosis in cutaneous T-cell lymphoma cell lines through the AKT pathway. J Invest Dermatol 126: 1641–1647 16645590 10.1038/sj.jid.5700322

[bib43] Rademaker-Lakhai JM, Beerepoot LV, Mehra N, Radema SA, van Maanen R, Vermaat JS, Witteveen EO, Visseren-Grul CM, Musib L, Enas N, van Hal G, Beijnen JH, Schellens JH, Voest EE (2007) Phase I pharmacokinetic and pharmacodynamic study of the oral protein kinase C beta-inhibitor enzastaurin in combination with gemcitabine and cisplatin in patients with advanced cancer. Clin Cancer Res 13: 4474–4481 17671132 10.1158/1078-0432.CCR-06-2912

[bib44] Rizvi MA, Ghias K, Davies KM, Ma C, Weinberg F, Munshi HG, Krett NL, Rosen ST (2006) Enzastaurin (LY317615), a protein kinase Cbeta inhibitor, inhibits the AKT pathway and induces apoptosis in multiple myeloma cell lines. Mol Cancer Ther 5: 1783–1789 16891464 10.1158/1535-7163.MCT-05-0465

[bib45] Rots MG, Pieters R, Peters GJ, van Zantwijk CH, Mauritz R, Noordhuis P, Willey JC, Hahlen K, Creutzig U, Janka-Schaub G, Kaspers GJ, Veerman AJ, Jansen G (1999) Circumvention of methotrexate resistance in childhood leukemia subtypes by rationally designed antifolates. Blood 94: 3121–3128 10556198

[bib46] Schwartz GK, Shah MA (2005) Targeting the cell cycle: a new approach to cancer therapy. J Clin Oncol 23: 9408–9421 16361640 10.1200/JCO.2005.01.5594

[bib47] Shaik MS, Chatterjee A, Jackson T, Singh M (2006) Enhancement of antitumor activity of docetaxel by celecoxib in lung tumors. Int J Cancer 118: 396–404 16052515 10.1002/ijc.21325 PMC2907249

[bib48] Sigmond J, Backus HH, Wouters D, Temmink OH, Jansen G, Peters GJ (2003) Induction of resistance to the multitargeted antifolate Pemetrexed (ALIMTA) in WiDr human colon cancer cells is associated with thymidylate synthase overexpression. Biochem Pharmacol 66: 431–438 12907242 10.1016/s0006-2952(03)00287-9

[bib49] Spalding AC, Watson R, Davis ME, Kim AC, Lawrence TS, Ben-Josef E (2007) Inhibition of protein kinase Cbeta by enzastaurin enhances radiation cytotoxicity in pancreatic cancer. Clin Cancer Res 13: 6827–6833 18006785 10.1158/1078-0432.CCR-07-0454

[bib50] Tabatabai G, Frank B, Wick A, Lemke D, von Kürthy G, Obermuller U, Heckl S, Christ G, Weller M, Wick W (2007) Synergistic antiglioma activity of radiotherapy and enzastaurin. Ann Neurol 61: 153–161 17212356 10.1002/ana.21057

[bib51] Tang JM, He QY, Guo RX, Chang XJ (2006) Phosphorylated Akt overexpression and loss of PTEN expression in non-small cell lung cancer confers poor prognosis. Lung Cancer 51: 181–191 16324768 10.1016/j.lungcan.2005.10.003

[bib52] Temmink OH, Prins HJ, van Gelderop E, Peters GJ (2007) The Hollow Fibre Assay as a model for *in vivo* pharmacodynamics of fluoropyrimidines in colon cancer cells. Br J Cancer 96: 61–66 17179993 10.1038/sj.bjc.6603507 PMC2360204

[bib53] Tonkinson JL, Marder P, Andis SL, Schultz RM, Gossett LS, Shih C, Mendelsohn LG (1997) Cell cycle effects of antifolate antimetabolites: implications for cytotoxicity and cytostasis. Cancer Chemother Pharmacol 39: 521–531 9118464 10.1007/s002800050608

[bib54] Tonkinson JL, Worzalla JF, Teng CH, Mendelsohn LG (1999) Cell cycle modulation by a multitargeted antifolate, LY231514, increases the cytotoxicity and antitumor activity of gemcitabine in HT29 colon carcinoma. Cancer Res 59: 3671–3676 10446980

[bib55] van Triest B, Loftus BM, Pinedo HM, Backus HH, Schoenmakers P, Telleman F, Tadema T, Aherne GW, van Groeningen CJ, Zoetmulder FA, Taal BG, Johnston PG, Peters GJ (2000) Thymidylate synthase expression in patients with colorectal carcinoma using a polyclonal thymidylate synthase antibody in comparison to the TS 106 monoclonal antibody. J Histochem Cytochem 48: 755–760 10820149 10.1177/002215540004800604

[bib56] van Triest B, Pinedo HM, van Hensbergen Y, Smid K, Telleman F, Schoenmakers PS, van der Wilt CL, van Laar JA, Noordhuis P, Jansen G, Peters GJ (1999) Thymidylate synthase level as the main predictive parameter for sensitivity to 5-fluorouracil, but not for folate-based thymidylate synthase inhibitors, in 13 nonselected colon cancer cell lines. Clin Cancer Res 5: 643–654 10100718

[bib57] Yu BZ, Zheng J, Yu AM, Shi XY, Liu Y, Wu DD, Fu W, Yang J (2004) Effects of protein kinase C on M-phase promoting factor in early development of fertilized mouse eggs. Cell Biochem Funct 22: 291–298 15338468 10.1002/cbf.1103

[bib58] Yu L, Orlandi L, Wang P, Orr MS, Senderowicz AM, Sausville EA, Silvestrini R, Watanabe N, Piwnica-Worms H, O'Connor PM (1998) UCN-01 abrogates G2 arrest through a Cdc2-dependent pathway that is associated with inactivation of the Wee1Hu kinase and activation of the Cdc25C phosphatase. J Biol Chem 273: 33455–33464 9837924 10.1074/jbc.273.50.33455

[bib59] Yuan A, Yu CJ, Shun CT, Luh KT, Kuo SH, Lee YC, Yang PC (2005) Total cyclooxygenase-2 mRNA levels correlate with vascular endothelial growth factor mRNA levels, tumor angiogenesis and prognosis in non-small cell lung cancer patients. Int J Cancer 115: 545–555 15704107 10.1002/ijc.20898

